# Association between radiation, glaucoma subtype, and retinal vessel diameter in atomic bomb survivors

**DOI:** 10.1038/s41598-019-45049-7

**Published:** 2019-06-14

**Authors:** Yoshiaki Kiuchi, Masahide Yanagi, Katsumasa Itakura, Ikuno Takahashi, Ayumi Hida, Waka Ohishi, Kyoji Furukawa

**Affiliations:** 1 Department of Ophthalmology and Visual Science, Hiroshima University, 1-2-3 Kasumi, Minami-ku, Hiroshima 734-8551 Japan; 2 Department of Clinical Studies, Radiation Effects Research Foundation (RERF), 5-2 Hijiyama Park, Minami-ku, Hiroshima 732-0815 Japan; 3 Biostatistics Center, Kurume University, 67 Asahi-machi, Kurume, Fukuoka 830-0011 Japan

**Keywords:** Epidemiology, Optic nerve diseases

## Abstract

We examined the relationship between glaucoma subtype and retinal vascular caliber as markers of ocular circulation. Subjects were Japanese atomic bomb survivors in Hiroshima and Nagasaki. After a screening examination, potential cases were subjected to further definitive examination. The diameters of central retinal artery and vein equivalents (CRAE and CRVE) on digitized retinal photographs were measured using an established method. Generalized linear regression analyses were used to examine the associations among vessel diameters, radiation exposure, and prevalence of glaucoma subtypes among the study subjects. We identified 196 cases of glaucoma (12%) based on optic disc appearance, perimetry results, and other ocular findings. The main subtypes were primary angle-closure glaucoma, primary open-angle glaucoma and normal-tension glaucoma (NTG). NTG was the dominant subtype (78%). NTG was negatively associated with CRAE and CRVE, and positively associated with radiation dose. CRVE was negatively associated with radiation dose and the association was unclear for CRAE. The smaller retinal vessel caliber in NTG patients than in subjects without glaucoma may indicate an association between ocular blood flow and the pathogenesis of NTG. However, significant relationships among vessel calibers, NTG and radiation exposure were not clear.

## Introduction

Glaucoma is one of the leading causes of blindness worldwide^1^. Typical glaucoma involves elevated intraocular pressure (IOP), which causes damage to structures in and around the optic nerve head; this leads to visual dysfunction^2^. Open-angle glaucoma without high IOP is known as normal-tension glaucoma (NTG); it is the predominant subtype in East Asia^3–5^. Cumulative evidence shows persistent progression of visual field loss in NTG, despite interventional reduction in IOP^6^. This may indicate that some factors other than IOP (e.g., insufficient blood supply in relation to systemic and/or retinal vascular disorders) play pivotal roles in glaucomatous optic neuropathy^7–12^.

We previously reported a correlation between the higher prevalence of NTG and radiation dose in Japanese atomic bomb survivors^13^, although the specific causal mechanism is unclear. Prior epidemiological studies have shown that radiation exposure at doses >0.5 Gy might be associated with an elevated risk of cardiovascular disease, particularly in atomic bomb survivors^14–16^; thus, radiation-related perturbed ocular circulation may be linked with NTG development among people with radiation exposure. The primary objective of this study was to evaluate subtype-specific associations of glaucoma with ocular vessel diameters and other potential risk factors for glaucoma, including radiation dose, among a clinical cohort of atomic bomb survivors.

## Results

Table 1 provides the baseline characteristics by glaucoma status in the right eyes of the 1,640 eligible subjects in the Adult Health Study (AHS) (36% men; mean age of 74.7 ± 6.5 years). Of 196 glaucoma cases (12%), NTG was the dominant subtype (78%). Mean ages of the subjects with glaucoma were 75.8 years for NTG, 76.6 years for primary open-angle glaucoma (POAG), and 76.5 years for primary angle closure glaucoma (PACG); these were slightly older than the 74.1 years for those without glaucomatous changes (non-glaucoma cases) (P < 0.01 for heterogeneity). The NTG subjects had much higher mean radiation dose (0.66 Gy) than subjects with POAG (0.27 Gy), PACG (0.18 Gy), or without glaucoma (non-glaucoma subjects) (0.43 Gy) (P < 0.01); however, NTG subjects had smaller values of both central retinal artery and vein equivalents (CRAE and CRVE) (123.1 and 190.5 μm, respectively) than any of the other groups (P < 0.01). Among other observations, the subjects with POAG appeared to exhibit a greater incidence of hypertension (68%) and dyslipidemia (40%); women were dominant (83%) among the subjects with PACG. The difference in the mean or proportion between the groups of NTG and the other types (POAG and PACG) was significant for low-density lipoprotein cholesterol (LDL) (p = 0.02), radiation dose (p < 0.01) and IOP (p < 0.01). Similar findings were observed in the left eyes (Table S1).

**Table 1:** Baseline characteristics (mean or percentage) by glaucoma subtype of the right eye among Adult Health Study subjects (N = 1,640), 2006–2008.

variable	(unit)	Glaucoma subtype (Right)				P^*^
		Normal	NTG	POAG	PACG	
		(1,444)	(153)	(25)	(18)	
Sex	(% Male)	36.0	41.8	52.0	16.7	0.05
Age at exam	(years)	74.1	75.8	76.6	76.5	<0.01
City at exam	(% Hiroshima)	57.1	53.6	40.0	50.0	0.29
Radiation dose	(Gy)	0.43	0.66	0.27	0.18	<0.01
Intraocular pressure (IOP)	(mmHg)	12.5	12.8	17.0	13.7	<0.01
CRAE	(μm)	131.5	123.1	125.3	133.7	<0.01
CRVE	(μm)	197.6	190.5	199.7	197.3	<0.01
Smoking status	(% Never smoker)	57.6	54.2	48.0	61.1	0.66
Hypertension	(%)	43.0	50.3	68.0	38.9	0.03
Dyslipidemia	(%)	32.5	28.1	40.0	33.3	0.59
Diabetes	(%)	26.7	27.5	36.0	11.1	0.05
Body mass index	(kg/m^2^)	23.0	22.6	22.7	23.0	0.62
Systolic blood pressure	(mmHg)	129.4	131.7	133.9	139.0	0.02
Diastolic blood pressure	(mmHg)	74.5	75.6	75.4	76.3	0.51
Total cholesterol	(mg/dL)	206.2	201.9	207.6	206.9	0.51
HDL cholesterol	(mg/dL)	58.3	57.2	56.5	57.0	0.76
LDL cholesterol	(mg/dL)	112.5	106.6	120.9	113.3	0.04
HbA1c	(%)	5.64	5.57	5.76	5.41	0.31
White blood cell count	(Count × 10^2^)	56.1	56.1	59.2	47.1	0.08
CRP	(μg/L)	0.20	0.23	0.27	0.17	0.79

Abbreviations: NTG, normal-tension glaucoma; POAG, primary open-angle glaucoma; PACG, primary angle-closure glaucoma; CRAE, central retinal artery equivalents; CRVE, central retinal vein equivalents; CRP, C-reactive protein.

^*^P-value for the null hypothesis of equal proportions or means across glaucoma subtypes by chi-squared test of homogeneity or ANOVA F-test.

Table 2 shows the estimated associations in multinomial logistic regression analysis for the glaucoma status (right eyes) of the subjects with retinal vessel diameters (CRAE and CRVE), radiation dose, and other adjusting factors. CRVE and CRAE were separately included in the analysis; the estimated associations for all but CRVE were based on the regression analysis with CRAE (which exhibited a better fit than that with CRVE). The analyses with categorical vessel diameters were performed with missing category indicators assigned for those with missing CRAE/CRVE; those with the continuous variables excluded those with missing categories (although including those missing—using an indicator—did not have a large impact on either estimates or P-values).

**Table 2:** Estimated associations in multinomial logistic regression analysis for the glaucoma status of the right eye among Adult Health Study subjects, 2006–2008.

variable (unit)		Glaucoma subtypes (Right)								
		NTG			POAG			PACG		
		OR	95%CI	P^*^	OR	95%CI	P^*^	OR	95%CI	P^*^
sex	Men		reference			reference			reference	
	Women	0.70	0.43 to 1.15	0.15	0.39	0.11 to 1.39	0.14	4.02	0.87 to 18.62	0.08
age (years)	<=70		reference			reference			reference	
	71–80	1.04	0.67 to 1.63	0.85	1.76	0.55 to 5.61	0.34	1.68	0.48 to 5.92	0.42
	81+	2.05	1.23 to 3.41	0.01	4.28	1.04 to 17.59	0.04	3.72	0.89 to 15.52	0.07
city	Hiroshima		reference			reference			reference	
	Nagasaki	1.03	0.7 to 1.5	0.89	1.11	0.43 to 2.83	0.83	1.50	0.52 to 4.3	0.45
Radiation dose (Gy)	<0.005		reference			reference			reference	
	0.005–0.2	0.85	0.46 to 1.57	0.61	0.31	0.05 to 1.71	0.18	2.86	0.88 to 9.22	0.08
	0.2–1	1.61	1.05 to 2.49	0.03	0.77	0.25 to 2.32	0.64	0.88	0.22 to 3.43	0.85
	1+	1.83	1.15 to 2.91	0.01	0.72	0.18 to 2.86	0.64	0.44	0.05 to 3.65	0.45
	continuous^†^	1.39	1.15 to 1.69	<0.01	0.77	0.32 to 1.82	0.54	0.44	0.13 to 1.54	0.20
IOP (mmHg)		1.05	0.99 to 1.12	0.12	1.60	1.39 to 1.84	<0.01	1.19	1.01 to 1.4	0.04
CRAE (μm)	<125	2.42	1.49 to 3.94	<0.01	2.62	0.74 to 9.21	0.13	2.90	0.57 to 14.78	0.20
	125–<135		reference			reference			reference	
	135+	0.80	0.45 to 1.43	0.45	1.33	0.33 to 5.38	0.69	3.36	0.67 to 16.82	0.14
	missing	1.84	1.05 to 3.23	0.03	1.61	0.36 to 7.17	0.53	1.79	0.28 to 11.36	0.54
	continuous^‡^	0.97	0.95 to 0.98	<0.01	0.98	0.94 to 1.01	0.19	1.02	0.98 to 1.05	0.33
CRVE (μm)	<190	1.50	0.95 to 2.35	0.08	1.09	0.31 to 3.78	0.89	2.50	0.64 to 9.8	0.19
	190–<205		reference			reference			reference	
	205+	0.76	0.45 to 1.29	0.31	1.29	0.36 to 4.54	0.70	3.03	0.71 to 13	0.14
	missing	1.29	0.73 to 2.31	0.38	2.09	0.5 to 8.7	0.31	0.57	0.06 to 5.76	0.63
	continuous^‡^	0.98	0.97 to 0.99	<0.01	1.01	0.98 to 1.04	0.45	1.01	0.98 to 1.03	0.61
smoking	never		reference			reference			reference	
	current	0.68	0.32 to 1.46	0.32	1.12	0.25 to 5.04	0.88	1.37	0.15 to 12.62	0.78
	past	0.89	0.53 to 1.5	0.67	0.47	0.12 to 1.83	0.28	1.91	0.52 to 6.96	0.33
Hypertension	No		reference			reference			reference	
	Yes	1.35	0.94 to 1.93	0.10	2.06	0.82 to 5.22	0.13	0.63	0.22 to 1.76	0.38
Dyslipidemia	No		reference			reference			reference	
	Yes	0.80	0.54 to 1.2	0.28	1.19	0.48 to 2.94	0.70	0.80	0.28 to 2.33	0.68
Diabetes	No		reference			reference			reference	
	Yes	0.98	0.56 to 1.71	0.93	2.60	0.89 to 7.55	0.08	0.00	0.0 to 0.0	<0.01
BMI (kg/m^2^)	<18.5	1.82	1.03 to 3.21	0.04	1.28	0.25 to 6.63	0.77	1.54	0.29 to 8.04	0.61
	18.5–<25		reference			reference			reference	
	25+	0.96	0.63 to 1.47	0.85	0.48	0.16 to 1.43	0.19	2.56	0.93 to 7.06	0.07
CRP (μg/dl)		1.09	0.84 to 1.42	0.53	1.29	0.8 to 2.1	0.30	0.89	0.2 to 4.02	0.88

Abbreviations: BMI, body mass index; CRAE, central retinal artery equivalents; CRVE, central retinal vein equivalents; IOP, intraocular pressure; CRP, C-reactive protein.

^*^P-value for the null hypothesis that the odds ratio is one by the Wald test.

^†^Odds ratio per unit increase in Gy.

^‡^Odds ratio per unit increase in μm.

The prevalence of each subtype tended to increase with aging. The prevalence of NTG significantly increased with reduction in CRAE (odds ratio (OR) per unit increase in μm = 0.97, 95% confidence interval (CI): 0.95 to 0.98, P < 0.01) and CRVE (OR per unit increase in μm = 0.98, 95% CI: 0.97 to 0.99, P < 0.01); NTG prevalence increased with radiation dose (OR per unit increase in Gy = 1.39, 95% CI: 1.15 to 1.69, P < 0.01). Associations of CRAE or CRVE with POAG or PACG were not as clear as those with NTG. The prevalence of POAG or PACG, in contrast to that of NTG, was lower for subjects who had received higher doses, with estimated ORs of 0.77 and 0.44, respectively; however, the associations were not statistically significant (P≥0.2). High IOP was associated with POAG and PACG; however, IOP level did not have a significant effect on NTG. Similar observations were obtained in the analysis for the left eye (Table S2).

**Figure 1:**
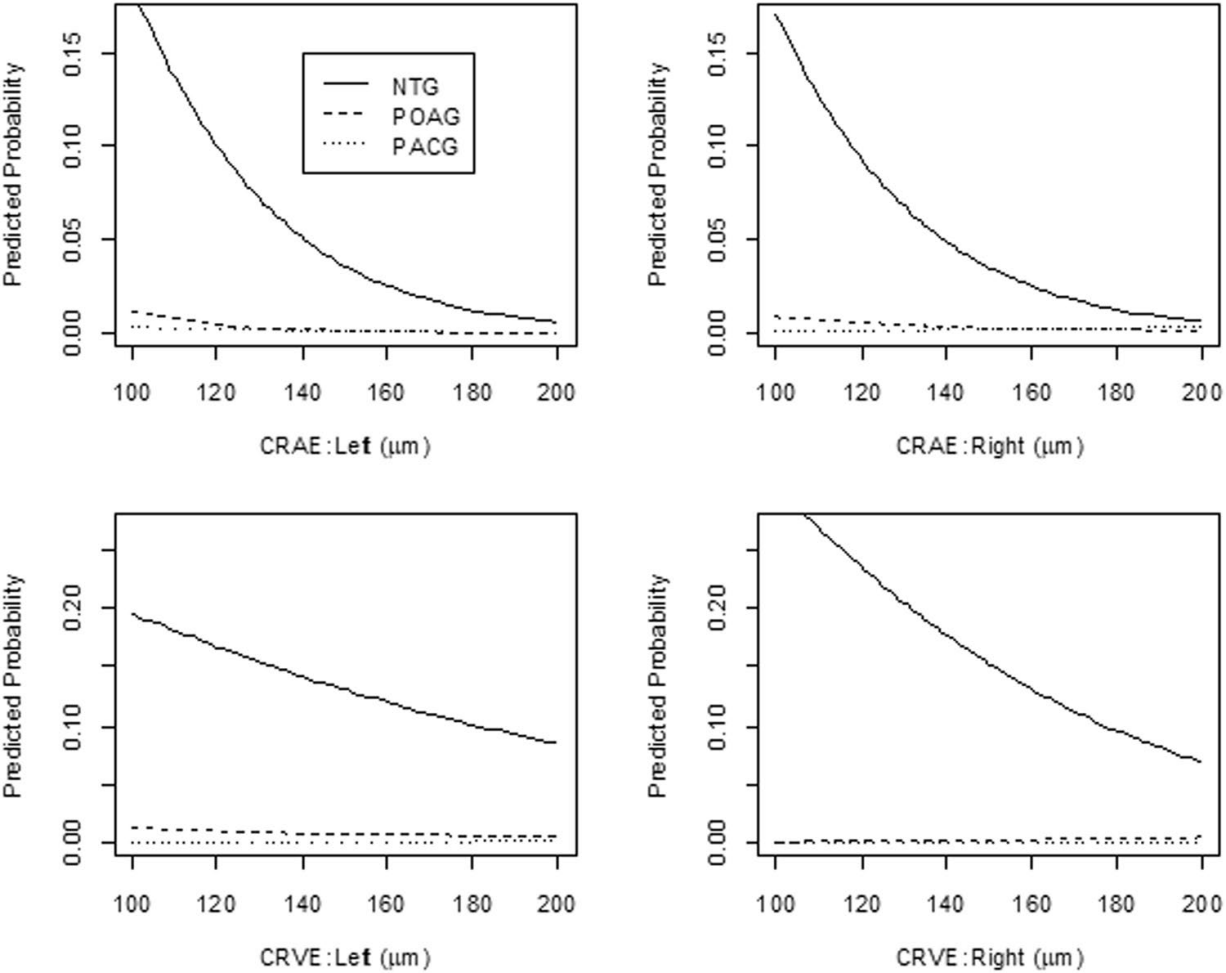
Estimated probabilities for each glaucoma type as a function of retinal vessel diameters among Adult Health Study subjects (N = 1,640), 2006–2008. The predicted probability of normal tension glaucoma tended to sharply decline with increasing central retinal artery equivalents and central retinal vein equivalents. Abbreviations: NTG, normal-tension glaucoma; POAG, primary open-angle glaucoma; PACG, primary angle-closure glaucoma.

Interpretation and comparison of OR estimates among the subtypes required some caution due to the considerable variability in baseline prevalence among the subtypes. Figure 1 illustrates the estimated prevalence of each glaucoma type as a function of retinal vessel diameters, when each considered predictor is at the reference level (as shown in Table 2) or the mean value. While estimated ORs were fairly comparable among the subtypes, the predicted probability of NTG tended to sharply decline with increasing CRAE and CRVE, reflecting a much higher background prevalence of NTG, compared with POAG and PACG.

Table 3 shows the estimated associations in linear regression analysis for the retinal vessel diameters with radiation dose and other adjustment factors. Here, data for both eyes were jointly analyzed using a linear mixed-effect model with a random intercept allowed to vary by individual. Diameters in CRVE tended to decline as dose increased, with an estimated mean change of −1.41 μm per 1 Gy radiation exposure (95% CI: −2.7 to −0.1, P = 0.03). The mean CRVE of those highly exposed to radiation exposure at 1 Gy or greater was 2.36 μm narrower than that of those with little exposure (<0.005 Gy (P = 0.08)); however, there was no statistical evidence for a significant quadratic term (P = 0.14) or threshold (P > 0.20). Table 3 also indicates that CRAE decreased with aging; notably, it was larger among women, compared with men (+2.78 μm, 95% CI: 0.8 to 4.7, P = 0.01), and was smaller among subjects in Nagasaki, compared with those in Hiroshima (−1.93 μm, 95% CI: −3.4 to −0.5, P = 0.01). Similarly, CRVE decreased with aging; it was larger among smokers (+6.79 μm, 95% CI: 3.1 to 10.5, P < 0.01) and was smaller among those in Nagasaki (−8.46 μm, 95% CI: −10.4 to −6.5, P < 0.01). In contrast to CRAE, CRVE was larger among subjects with hypertension (+2.23 μm, 95% CI: 0.3 to 4.2, P = 0.02) and those with diabetes (+3.02 μm, 95% CI: 0 to 6.0, P = 0.05). IOP was negatively associated with CRAE (−0.26 μm per increase of IOP in mmHg, 95% CI: −0.5 to 0.0, P = 0.02).

**Table 3:** Estimated associations in linear regression analysis for the retinal vessel diameter (CRAE and CRVE) of both left and right eyes among Adult Health Study subjects, 2006–2008.

variable (unit)		CRAE			CRVE		
		estimate	95%CI	P^*^	estimate	95%CI	P^*^
sex	Men		reference			reference	
	Women	2.78	0.8 to 4.7	0.01	−0.64	−3.3 to 2	0.64
age (years)	<=70		reference		reference		
	71–80	−0.75	−2.3 to 0.8	0.34	−0.42	−2.6 to 1.7	0.70
	81+	−4.22	−6.3 to −2.1	<0.01	−5.58	−8.4 to −2.8	<0.01
city	Hiroshima	reference		reference			
	Nagasaki	−1.93	−3.4 to −0.5	0.01	−8.46	−10.4 to −6.5	<0.01
Radiation dose (Gy)	<0.005		reference		reference		
	0.005–0.2	−0.97	−3.1 to 1.1	0.36	−1.11	−4 to 1.7	0.45
	0.2–1	0.33	−1.4 to 2	0.70	−0.42	−2.7 to 1.9	0.72
	1+	−0.35	−2.3 to 1.6	0.72	−2.36	−5 to 0.3	0.08
	continuous^†^	−0.33	−1.3 to 0.6	0.49	−1.41	−2.7 to −0.1	0.03
IOP (mmHg)	−0.26	−0.5 to −0.03	0.02	−0.21	−0.5 to 0.1	0.16	
smoking	never		reference		reference		
	current	2.50	−0.2 to 5.2	0.07	6.79	3.1 to 10.5	<0.01
	past	0.35	−1.7 to 2.4	0.74	1.48	−1.4 to 4.3	0.31
Hypertension	No		reference		reference		
	Yes	−0.96	−2.4 to 0.5	0.18	2.23	0.3 to 4.2	0.02
Dyslipidemia	No		reference		reference		
	Yes	−0.04	−1.5 to 1.5	0.96	−0.81	−2.9 to 1.2	0.44
Diabetes	No		reference		reference		
	Yes	0.66	−1.5 to 2.9	0.56	3.02	0 to 6	0.05
BMI (kg/m^2^)	<18.5	1.52	−1 to 4	0.24	−0.66	−4.1 to 2.8	0.71
	18.5–<25	reference		reference			
	25+	−0.70	−2.3 to 0.9	0.39	1.56	−0.6 to 3.7	0.16
CRP (μmg)	−0.37	−2 to 1.3	0.66	−0.08	−1.8 to 1.6	0.92	

Abbreviations: CRAE, central retinal artery equivalents; CRVE, central retinal vein equivalents; BMI, body mass index; IOP, intraocular pressure; SBP, systolic blood pressure; DBP, diastolic blood pressure; HDL, high-density lipoprotein cholesterol; LDL, low-density lipoprotein cholesterol; CRP, C-reactive protein.

^*^P-value for the null hypothesis that the parameter is zero by the t-test.

^†^Mean change in the retinal vessel diameter (μm) per unit increase in Gy.

There was no significant association between the level of visual field (VF) and radiation dose, while the vessel diameter was significantly and negatively associated with the stage of VF grading for both CRAE (P = 0.01 and 0.05 for right and left, respectively) and CRVE (P < 0.01 for both right and left eyes). There was, however, no evidence of a significant difference in the strength of these associations based on the level of radiation exposure.

## Discussion

This is the first report to explore the potential contribution of perturbed ocular circulation to development of radiation-related NTG, using retinal images obtained from Japanese atomic bomb survivors. We found that the prevalence of NTG was significantly higher among the subjects with narrower retinal vessels (with estimated ORs per unit increase of diameter in μm of 0.97 [95% CI: 0.95 to 0.98] and 0.98 [95% CI: 0.97 to 0.99], for CRAE and CRVE, respectively (Table 2)). This relationship might partly be explained by the negative association between radiation and retinal vessels (with an estimated reduction of 1.41 μm in venular caliber per 1 Gy irradiation in eye [95% CI: −2.7 to −0.1]) (Table 3). Due to the much lower numbers of cases of POAG (25) and PACG (18) (Table 1), the associations related to these subtypes were not as clear as those related to NTG (Table 2, Fig. 1).

In the present study, a total of 196 right eyes (12.0%) and 207 left eyes (12.6%) among 1640 total pairs of eyes had glaucoma such as NTG, POAG, or PACG. The prevalence of POAG in right and left eyes was 11.3% and 10.9%, respectively. Of these POAG cases, proportions of NTG were 87.1% in the right eye and 86.0% in the left eye. The prevalence of PACG was 1.3% in the right eye and 1.5% in the left eye.

For comparison, we used the Tajimi Study, a population-based study conducted between 2000 and 2001 at Tajimi City in central Japan^3,17^. In that study, the prevalence of all glaucoma in subjects in their 70’s was 10.5%, and that of PACG was 1.4%. Additionally the proportion of overall NTG among open-angle glaucoma was 92.3%. Importantly, the prevalence of PACG in the present study was very similar to that in the Tajimi Study^3,17^. The prevalence of POAG (mean age 76.6 years) in the present study (11.3% in right eyes and 10.9% in left eyes) were slightly higher than those in the Tajimi study (8.2% in patients age 70–79). The prevalence of NTG in the Tajimi study in patients in their 70’s was not reported; however, based on the total NTG proportion, the calculated NTG prevalence in patients in their 70 s was 7.5%. The high prevalence of NTG in the present study (9.3%–9.8%) may have led to the high prevalence of POAG that we observed.

An earlier study indicated an effect of radiation from atomic bombs (0–4 Gy) on the prevalence of NTG, with estimated OR at 1 Gy of 1.31 [95% CI: 1.11 to 1.53]; no significant association was found for other types^13^. NTG risk, driven by retinal arteriolosclerosis related to radiation damage, is plausible. Uncertainties associated with nonparticipation (59% participation) might have hindered proper interpretation. However, the intensive bias check showed that the expected association for participants and nonparticipants combined should be even larger than the positive dose-response slope that was found in that study^13^. In other exposed populations, as a complication of high-dose and high dose-rate radiotherapy to the eye, neovascular glaucoma has been found in 7–48% of patients^18–23^. Doses of radiation >10 Gy obstruct microcirculation due to plaque formation, reducing ocular blood flow in the eye. This leads to neovascularization in the iris or angle, which results in high IOP. Notably, relatively low doses of radiation have not resulted in excess ocular hypertension in radiation workers; however, there are few available studies^23–25^. Howell *et al*. reported that sub-lethal γ-radiation protected retinal ganglion cells in DBA/2 J mice from the onset of glaucoma^26^.

Clarification of disease background could provide better understanding of radiation-related NTG. Our results showed a correlation between narrower retinal vessels and glaucoma, consistent with other reports^11,27–33^; it remains whether the change in retinal vessel diameter is a cause or result of the glaucoma development. Chan *et al*. suggested that insufficient blood supply may contribute to NTG development as a result of damage to retinal ganglion cells^34^. Conversely, Kawasaki *et al.*^11^ reported that retinal arteriolar narrowing quantitatively measured by retinal photographs is associated with the long-term risk of open-angle glaucoma. They speculated that retinal arteriolar narrowing might result from decreased oxygen demand after the loss of retinal ganglion cells, which is recognized as an early pathologic change of open angle glaucoma. We may be able to apply these hypotheses to our POAG and NTG subjects, but it is difficult to explain the reason why PACG subjects had retinal vessel caliber similar to that of the normal subject group in the present study. Among glaucoma subjects, only the incidence of NTG increased with radiation dose. If radiation exposure actually damages retinal ganglion cells directly, prevalence of all glaucoma subtypes should increase with radiation dose. Thus, we suspect that radiation does not cause direct harm to retinal ganglion cells. A prospective study of both retinal vessel diameter and retinal blood flow before and after radiation exposure might be useful, as would an assessment of the relationship between glaucoma prevalence and radiation dose.

There is, however, a discrepancy in the strength of association between arterioles and venules with retinal nerve fiber lesion or NTG: our data showed associations of NTG with arterioles and venules; some prior studies have indicated stronger associations with venules^30–32^, while others have indicated stronger associations with arterioles^11,33^. This may be explained by the complex interactions between various mediators for vasodilation and vasoconstriction on arterioles and venules. Narrower venular caliber may potentially indicate venous congestion and cytotoxic damage, with subsequent secondary constriction of arteriole^35,36^.

Our data also showed that elderly people had reduced CRAE/CRVE; notably, CRVE was apparently wider in subjects who were smokers, and who had hypertension or diabetes, in agreement with the evidence from elsewhere^37,38^. CRVE is thought to be wider due to reduction in vascular reactivity under diabetic conditions, or due to smoking-induced enhancement in nitric oxide production, potassium channel activation, and possible tissue degeneration^39^. In contrast, the underlying mechanism of narrowing of CRVE (associated with radiation and aging in our data) has not been determined; however, ischemic damage to the optic disc through dysregulation of endothelin (ET) may be involved. ET-1 is a pro-inflammatory peptide that activates monocyte chemoattractant protein-1 (MCP-1). ET-1 has been shown to contribute to microvessel dysfunction after exposure to ionizing radiation in a rat model^40^. MCP-1 is a monocyte-specific chemoattractant and activator; its expression has been detected in a variety of proinflammatory conditions, including atherosclerosis^41^. Past studies have shown elevated levels of ET-1 and MCP-1 among NTG patients^42–44^, as well as ET-1-induced optic nerve ischemia in a rabbit model^45^. Dysregulation of the ET may be attributable to pathological processes causing NTG through proinflammatory signaling. Our current analysis did not find any relationship between changes in retinal vessel diameters and the C-reactive protein (CRP) of inflammatory marker; although a prior survey suggested that radiation exposure can lead to proinflammatory status in atomic bomb survivors^46,47^, which may result in development of radiation-related NTG.

Our current data indicated that the means of CRAE and CRVE were significantly narrower among subjects in Nagasaki than among those in Hiroshima. While this might have been affected by unadjusted confounding, further studies are required to identify a factor that could clearly explain the observed difference. No such city difference was observed, however, in the prevalence of glaucoma with adjustment for CRAE or CRVE.

The strength of this study is that we diagnosed glaucoma and measured vascular dimensions in a well-defined cohort with a wide range of reasonably accurate dose estimates. A well-established computer imaging program was used to acquire the retinal vascular measurements by a trained grader who was blinded to the participant characteristics.

However, there are several limitations associated with the present study. First, as noted, the cross-sectional nature of our data does not provide temporal information regarding these associations in order to determine whether narrowing of the retinal vessels is antecedent or consequent to glaucomatous optic neuropathy. Second, we cannot exclude the possibility that the associations observed in this study are due to residual confounders such as disc shape or axial length^48,49^. Third, there may be a selection bias due to the exclusion of subjects with either no fundus photos or inadequate-quality photographs for assessment of retinal vessel caliber. Our results may not be directly applicable to the general population, as atomic bomb survivors exposed to radiation were included in this analysis. Fourth, a mortality bias should also be noted. Individuals with a high level of radiation exposure may have died before they could be included in this study. Notwithstanding these limitations, this is the investigation to evaluate associations between vascular dimensions and radiation-related NTG.

In conclusion, smaller retinal vessel caliber derived from alterations in ocular microcirculation may contribute to the development of NTG in atomic bomb survivors. However, we could not show the mechanism how the radiation exposure increased the prevalence of NTG in our subjects clearly.

## Methods

### Subjects

**Figure 2:**
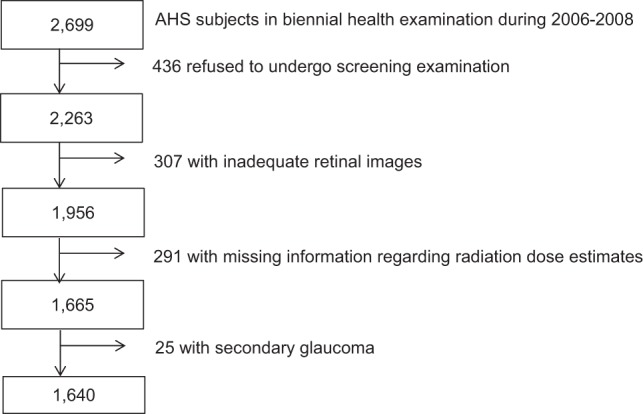
Data sample exclusion. Abbreviations: AHS, Adult Health Study of the Japanese atomic bomb survivors.

The AHS is a cohort study that includes a biannual health examination program investigating the effects of whole-body irradiation on human health among Japanese atomic bomb survivors in Hiroshima and Nagasaki; it has been active since 1958. Comprehensive ocular examinations were performed during the period from 2006 to 2008 with written informed consent and ethical approval from the Radiation Effects Research Foundation (RERF) Ethics Committee for this study, consistent with the provisions of the Declaration of Helsinki. Of 2,699 AHS subjects nominated in the current examination cycle, 436 subjects refused to participate this screening. After excluding 307 subjects with retinal images that were inadequate for evaluating vessel diameters in eyes, 291 subjects lacking eye radiation dose estimates, and 25 subjects with secondary glaucoma, a total of 1,640 subjects were evaluated in this study (Fig. 2). We conducted screening tests and definitive examinations for glaucoma in both eyes of each subject.

### Screening tests and definitive examinations for glaucoma

Our study consisted of ophthalmological screening and further definitive examinations, in accordance with the established method used by Tajimi Study;^3^ the details are described elsewhere^13^. Glaucoma specialists reviewed initial screening records, and then conducted further definitive examinations among selected patients with ocular disease.

The primary screening tests involved measurement of IOP, fundus photograph, and a visual field test. The examiners performed all tests blindly with respect to the radiation doses. IOP was measured three times with a non-contact tonometer (Topcon CT-90A; Topcon Corporation, Tokyo, Japan), and the mean value was used for each subject. With the pupil undilated, digital color photographs of the fundus were obtained using a digital fundus camera (Topcon NW6S; Topcon Corporation) with angles of 30° and 45°. The visual field was screened using the Frequency Doubling Technology C-20-1 screener (Carl Zeiss Meditec Inc., Dublin, CA, USA).

In the definitive examination, the following evaluations were performed: a slit-lamp biomicroscopic examination, Goldmann applanation tonometry, a visual field test using a Humphrey Field Analyzer (Carl Zeiss Meditec Inc.) 30-2 Swedish Interactive Threshold Algorithm standard program, gonioscopy using a Goldmann 2-mirror lens, and optic nerve head evaluation. When gonioscopy showed no contraindication, the pupil was dilated with 0.5% tropicamide and 0.5% phenylephrine hydrochloride. Following dilation, stereoscopic disc photographs (Topcon TR 80; Topcon RM-8000; Topcon Corporation). If a glaucomatous disc change or nerve fiber defect was found, and if the hemifield-based visual field abnormality was compatible with the optic disc appearance or nerve fiber defect, the eye was diagnosed with glaucoma. The results of VF test were graded by mean deviation (MD) obtained from the Humphrey visual field test (normal, early, moderate, or severe). Early glaucoma was defined as eyes with visual field loss with an MD ≥ −6 dB, moderate glaucoma as eyes with an MD of −6 to −12 dB, and severe glaucoma as those with an MD < −12 dB^50^. The cup-to-disc ratio and rim width were obtained by computer software-assisted fundus photoplanimetry^51^.

Eyes were diagnosed with POAG if the eye had an IOP > 21 mmHg by Goldmann applanation tonometry and an open angle of Shaffer grade ≥ 3. Eyes which met the criteria for POAG except that of IOP (i.e. IOP of ≤21 mmHg) were diagnosed with POAG (NTG). POAG (NTG) was described as NTG. Eyes with glaucomatous optic disc changes that also had an occludable angle of Shaffer grade ≤ 2 were diagnosed with PACG^17^.

### Measurement of the retinal vascular caliber

Digital fundus color photographs taken through undilated pupils, using a fundus camera at the screening examination at RERF, were used for the analysis, as described elsewhere^37^. An established method^37,52,53^, using a semi-automated computer imaging program (Retinal Analysis-IVAN, University of Wisconsin, Madison, WI, USA), was applied to obtain the central retinal artery and vein equivalents (CRAE and CRVE, respectively) by a trained grader who was masked to the subjects’ characteristics under excellent reproducibility; the intraclass correlation coefficient was high (>0.90).

### Systemic assessment of potential confounders

Potential confounders included age, sex, systolic and diastolic blood pressures, and the following factors. White blood cell count, CRP, high density lipoprotein cholesterol, and low-density lipoprotein cholesterol were measured using our defined quality control. Histories of hypertension, diabetes, and dyslipidemia were defined as described elsewhere^54^. Body mass index (BMI) was defined as the weight divided by the height squared. The radiation dose to the eye received by each participant was estimated based on the updated dosimetry system (DS02R1) that considers physical location and orientation at the time of the bombing, as well as shielding by terrain and organ shielding by the body^55,56^. The dose to an individual is the sum of (γ ray dose) + (10× the smaller neutron dose).

### Statistical methods

A multinomial logistic regression model was used to describe associations between the retinal vessel diameter and a nominal response variable of glaucoma status among the AHS subjects. Let *Y* be a categorical response with four categories of the glaucoma status (1: non-glaucoma, 2: NTG, 3: POAG, 4: PACG) and ${\pi }_{j}({\bf{x}})=P(Y=j|{\bf{x}})$, *j* = 1, 2, 3, 4, where **x** = {*x*_1_, *x*_2_, …, *x*_*p*_} is a vector of *p* explanatory variables, with $\sum _{j}{\pi }_{j}({\bf{x}})=1$. Then, the counts at the four categories of *Y* were treated as multinomials with probabilities {${\pi }_{1}({\bf{x}}),\,{\pi }_{2}({\bf{x}}),{\pi }_{3}({\bf{x}}),{\pi }_{4}({\bf{x}})$}. The effects of **x** on each glaucoma type were described by the logit of the *j*-th type (*j* = 2, 3, 4) to the non-glaucoma status (*j* = 1):

$$\mathrm{log}\,\frac{{\pi }_{j}({\bf{x}})}{{\pi }_{1}({\bf{x}})}={{\rm{\alpha }}}_{j}+{\boldsymbol{\beta }}{\text{'}}_{j}{\bf{x}},j=2,3,4,$$

where each component ${\beta }_{j,k}$ of the parameter vectors {${{\boldsymbol{\beta }}}_{j}=({\beta }_{j,1},\ldots {\beta }_{j,p});\,j=2,3,4\}$ is the log ratio of the odds that one has the *j*-th glaucoma type (instead of non-glaucoma) for the given level, compared with the reference level of a categorical variable, or for each unit increase of a continuous variable. For **x** in the current analysis, we considered categorical variables of sex, city (Hiroshima or Nagasaki), age at examination (<=70, 71–80, or 81+), smoking status (Never, Past, Current, or Unknown), BMI (<18.5, 18.5 to <25, or ≥25 kg/m^2^), and disease status of hypertension, dyslipidemia, and diabetes, as well as continuous variables (centered at the mean values) of IOP (mmHg) and CRP (μg/L). Radiation dose was included in analysis either categorically (<0.005, 0.005–0.2, 0.2–1, or >1 Gy) or as a continuous variable. Similarly, CRAE and CRVE measurements (in μm) were considered in the analysis either as continuous variables (centered at the mean) or categorically with categories based on equally-spaced percentiles: (<125, 125–135, or >135 μm) for CRAE and (<190, 190–205, or >205 μm) for CRVE. Given a moderate correlation between CRAE and CRVE (with correlation coefficients of 0.40–0.47, P < 0.01 by Pearson’s product moment correlation test), the effects of these variables were examined separately. Analyses were conducted separately for left and right eyes.

Alternatively, we conducted normal linear regression to examine how retinal vessel diameters varied by radiation exposure and other relevant factors among the study subjects. In addition to separate analyses for the right and left eyes, linear mixed-effect regression analyses with a random intercept allowed to vary by individual were conducted with combined data for both eyes to adjust for within-subjects correlation between the eyes^57^.

Confidence intervals and tests for significance for estimated associations were based on the Wald method. Approximately 20% of the 1,640 subjects had measurements of CRAE or CRVE missing in either the right or left eye, with a higher missing rate among the elderly (20.8% (≤70 years) vs. 40.5% (>70 years) for CRAE; 17.5% (≤70 years) vs. 37.3% (>70 years) for CRVE); the proportion of those missing data did not vary according to glaucoma status. Analyses either excluded those with missing values or included them with a missing group indicator for CRAE or CREV. The adjustment factors of IOP or CRP also involved some missing values for approximately 5% of the subjects; these were excluded in the main analysis. All reported *P* values were based on two-sided tests of significance. The model fitting and statistical testing used the multinom function of the package “nnet” for the multinomial logistic regression and the lmer function of the package “lme4” for the linear mixed effect regression in R (version 3.2.1; The R Project for Statistical Computing).

## Supplementary information

Supplementary tables

## Data Availability

There are no linked research data sets for this submission because the authors do not have permission to share data.
