# Supplementary material for: Association between radiation, glaucoma subtype, and retinal vessel diameter in atomic bomb survivors
Source: Sci Rep. 2019 Jun 14;9:8642. doi: 10.1038/s41598-019-45049-7 (PMC6570769; doi:10.1038/s41598-019-45049-7)
Supplement: Supplementary file 1 — Supplementary tables [file 41598_2019_45049_MOESM1_ESM.pdf]

Supplementary tables

Association between radiation, glaucoma subtype, and retinal vessel diameter in atomic bomb survivors

Authors:

Yoshiaki Kiuchi*, MD, PhD,[1] Masahide Yanagi, MD, PhD,[1] Katsumasa Itakura, MD, PhD,[1]

Ikuno Takahashi, MD, PhD,[2] Ayumi Hida, MD, PhD,[2] Waka Ohishi, MD, PhD,[2]

Kyoji Furukawa, PhD,[3]

[1] Department of Ophthalmology and Visual Science, Hiroshima University

1-2-3 Kasumi, Minami-ku, Hiroshima, 734-8551, Japan

[2] Department of Clinical Studies, Radiation Effects Research Foundation (RERF)

5-2 Hijiyama Park, Minami-ku, Hiroshima, 732-0815, Japan

[3] Biostatistics Center, Kurume University

67 Asahi-machi, Kurume, Fukuoka, 830-0011, Japan

Supplementary materials

Table S1. Baseline characteristics (mean or percentage) by glaucoma subtype of the left eye among the Adult Health Study subjects (N=1,640), 2006-2008

variable	(unit)	Glaucoma subtype (Left)				P*
		Normal	NTG	POAG	PACG	
		(1,444)	(153)	(25)	(18)	
**Sex**	**(% Male)**	36.5	37.7	54.2	14.3	0.04
**Age at exam**	**(years)**	74.1	75.5	76.5	76.9	0.01
**City at exam**	**(% Hiroshima)**	56.7	56.2	41.7	52.4	0.51
**Radiation dose**	**(Gy)**	0.44	0.58	0.19	0.19	0.01
**Intraocular pressure (IOP)**	**(mm Hg)**	12.9	13.2	17.0	14.0	<0.01
**CRAE**	**(μm)**	128.1	121.3	117.8	121.8	<0.01
**CRVE**	**(μm)**	197.4	192.3	189.0	199.3	0.01
**Smoking status**	**(% Never smoker)**	56.9	59.9	45.8	66.7	0.47
**Hypertension**	**(%)**	42.8	53.7	62.5	33.3	0.01
**Dyslipidemia**	**(%)**	32.3	29.6	33.3	42.9	0.67
**Diabetes**	**(%)**	26.9	25.3	33.3	19.0	0.07
**Body mass index**	**(kg/m^2)**	22.9	22.7	22.9	22.9	0.84
**Systolic blood pressure**	**(mm Hg)**	129.3	131.8	135.8	137.2	0.02
**Diastolic blood pressure**	**(mm Hg)**	74.5	75.6	75.8	75.5	0.54
**Total cholesterol**	**(mg/dL)**	206.4	199.8	210.4	204.4	0.11
**HDL cholesterol**	**(mg/dL)**	58.1	59.3	57.9	56.7	0.74
**LDL cholesterol**	**(mg/dL)**	113.0	102.5	117.3	116.0	<0.01
**HbA1c**	**(%)**	5.64	5.55	5.81	5.50	0.25
**White blood cell count**	**($Count \times 10^2$)**	56.1	55.9	61.7	49.0	0.06
**CRP**	**(μg/L)**	0.21	0.20	0.15	0.15	0.93

Abbreviations: NTG, normal tension glaucoma; POAG, primary open-angle glaucoma; PACG, primary angle closure glaucoma; CRAE, central retinal artery equivalents; CRVE, central retinal vein equivalents; CRP, C-reactive protein

* P-value for the null hypothesis of equal proportions or means across glaucoma subtypes by chi-squared test of homogeneity or ANOVA F-test.

Table S2. Estimated associations in multinomial logistic regression analysis for the glaucoma status of the left eye among the Adult Health Study subjects, 2006-2008

variable (unit)		Glaucoma subtypes (Left)								
		NTG			POAG			PACG		
		OR	95%CI	P	OR	95%CI	P	OR	95%CI	P
sex	Men		reference			reference			reference	
	Women	0.73	0.45 to 1.2	0.22	0.53	0.13 to 2.24	0.39	4.55	0.97 to 21.41	0.06
age (years)	<=70		reference			reference			reference	
	71-80	1.25	0.81 to 1.91	0.31	2.74	0.81 to 9.22	0.10	1.72	0.51 to 5.84	0.39
	81+	1.76	1.07 to 2.92	0.03	3.45	0.81 to 14.67	0.09	3.58	0.96 to 13.29	0.06
city	Hiroshima		reference			reference			reference	
	Nagasaki	0.89	0.62 to 1.28	0.52	1.03	0.39 to 2.68	0.96	1.16	0.43 to 3.09	0.77
Radiation dose (Gy)	<0.005		reference			reference			reference	
	0.005-0.2	1.03	0.6 to 1.79	0.91	1.11	0.34 to 3.56	0.86	2.15	0.71 to 6.54	0.18
	0.2-1	1.44	0.95 to 2.2	0.09	0.50	0.13 to 1.86	0.30	0.81	0.24 to 2.7	0.73
	1+	1.39	0.87 to 2.22	0.17	0.40	0.08 to 2.1	0.28	0.36	0.04 to 2.91	0.34
	continuous†	1.26	1.03 to 1.54	0.02	0.44	0.14 to 1.37	0.16	0.47	0.15 to 1.45	0.19
IOP (mmHg)		1.04	0.98 to 1.11	0.19	1.43	1.25 to 1.63	<0.01	1.15	0.99 to 1.34	0.07
CRAE (μm)	<125	1.71	1.1 to 2.64	0.02	11.96	1.49 to 95.74	0.02	2.79	0.76 to 10.25	0.12
	125 - <135		reference			reference			reference	
	135+	0.56	0.31 to 1.01	0.05	1.49	0.09 to 25.21	0.78	0.74	0.12 to 4.55	0.74
	missing	0.97	0.56 to 1.66	0.91	9.33	1.06 to 81.92	0.04	1.44	0.3 to 6.79	0.65
	continuous‡	0.96	0.95 to 0.98	<0.01	0.95	0.91 to 0.99	0.01	0.97	0.93 to 1.01	0.10
CRVE (μm)	<190	0.97	0.69 to 1.37	0.87	0.50	0.06 to 3.85	0.50	0.73	0.13 to 4.15	0.72
	190 - <205		reference			reference			reference	
	205+	1.56	1 to 2.44	0.05	1.10	0.36 to 3.36	0.86	3.33	0.68 to 16.2	0.14
	missing	0.90	0.54 to 1.49	0.69	0.75	0.19 to 2.86	0.67	3.97	0.79 to 19.86	0.09
	continuous‡	0.99	0.98 to 1	0.03	0.99	0.97 to 1.01	0.32	1.01	0.99 to 1.04	0.38
smoking	never		reference			reference			reference	
	current	0.56	0.26 to 1.2	0.14	0.93	0.17 to 5.04	0.93	1.41	0.15 to 13.59	0.77
	past	0.64	0.38 to 1.09	0.10	0.48	0.11 to 2.12	0.34	1.53	0.44 to 5.37	0.50
Hypertension	No		reference			reference			reference	
	Yes	1.64	1.15 to 2.33	0.01	2.04	0.82 to 5.1	0.13	0.48	0.18 to 1.29	0.15
Dyslipidemia	No		reference			reference			reference	
	Yes	0.79	0.54 to 1.16	0.23	0.85	0.33 to 2.21	0.74	1.29	0.5 to 3.29	0.60
Diabetes	No		reference			reference			reference	
	Yes	0.93	0.53 to 1.63	0.79	2.48	0.82 to 7.53	0.11	0.00	0 to 0	<0.01
BMI (kg/m^2)	<18.5	2.64	1.55 to 4.51	<0.01	2.02	0.39 to 10.44	0.40	1.45	0.29 to 7.24	0.65
	18.5 - <25		reference			reference			reference	
	25+	1.07	0.71 to 1.61	0.74	0.60	0.2 to 1.79	0.36	2.02	0.77 to 5.26	0.15
CRP (μg/dl)		0.96	0.65 to 1.41	0.83	0.40	0.04 to 3.58	0.41	0.75	0.13 to 4.13	0.74

Abbreviations: BMI, body mass index; CRAE, central retinal artery equivalents; CRVE, central retinal vein equivalents; IOP, intraocular pressure; CRP, C-reactive protein

* P-value for the null hypothesis that the odds ratio is one by the Wald test.

† Odds ratio per unit increase in Gy.

‡ Odds ratio per unit increase in μm.
